# Should Sinus of Valsalva be Replaced in Patients with Dilated Ascending Aorta and Aortic Valve Diseases?

**DOI:** 10.21470/1678-9741-2018-0093

**Published:** 2018

**Authors:** Salih Salihi, Emir Cantürk, Cengiz Köksal, Hızır Mete Alp

**Affiliations:** 1 Department of Cardiovascular Surgery, Okan University, Medicine Faculty Hospital, Istanbul, Turkey.; 2 Department of Cardiovascular Surgery, Bezmialem Vakıf University, Medical Faculty, Istanbul, Turkey.

**Keywords:** Aortic Aneurysm, Aortic Aneurysm, Thoracic/Surgery, Aorta/Surgery, Aortic Valve/Abnormalities, Bicuspid Aortic Valve

## Abstract

**Introduction:**

The aim of this study is to investigate the change in the dimension of sinus of Valsalva in patients who underwent supracoronary ascending aorta replacement with aortic valve replacement.

**Methods:**

A total of 81 patients who underwent supracoronary ascending aorta replacement with aortic valve replacement were included. Ten of 81 patients died during the follow-up. The patients were divided into three groups according to the aortic valve diseases. Group I (n=17) included patients with bicuspid valves, group II (n=30) included patients with stenotic degenerative valves, and patients with aortic regurgitation constituted group III (n=24). In preoperative and follow-up periods, the sinus of Valsalva diameter of the patients was evaluated by echocardiographic examination. The mean age was 54.1±15.1 years. Twenty-eight (34.6%) patients were female and 12 (14.8%) patients were in New York Heart Association functional class III.

**Results:**

There was no early mortality. Late mortality was developed in 10 (12.4%) patients, 8 (9.9%) due to non-cardiac reasons. Late follow-up was obtained in 71 patients with a mean of 60±30.1 months postoperatively. During follow-up, the increase in the diameter of the sinus of Valsalva was significant in Group I (*P*<0.01), while in Group II and III it was insignificant (*P*>0.05).

**Conclusion:**

To avoid the risks associated with sinus of Valsalva dilatation, it is reasonable to replace the sinus of Valsalva in the setting of aortic valve replacement and ascending aorta replacement for bicuspid aortic valve with a dilated ascending aorta and relatively normal sinuses of Valsalva in young patients.

**Table:** 

Abbreviations, acronyms & symbols	
**AAA**	**= Ascending aortic aneurysms**
**AAR**	**= Ascending aorta replacement**
**AVR**	**= Aortic valve replacement**
**BAV**	**= Bicuspid aortic valve**
**NCSS**	**= Number Cruncher Statistical System**
**NYHA**	**= New York Heart Association**

## INTRODUCTION

The prognosis of aortic valve diseases with ascending aortic aneurysms (AAA) varies according to the underlying etiology. The surgical strategy often depends on the aortic valve disease. Procedures include supracoronary ascending aorta replacement (AAR) with aortic valve replacement (AVR) and composite aortic valve graft replacement (Bentall-De Bono). It is often believed that the aortic aneurysm seen along with degenerative aortic stenosis is due to post-stenotic dilatation^[1]^. Valve intervention is necessary when aortic regurgitation is seen along with AAA. In the past, supracoronary AAR with AVR or composite aortic valve graft replacement was commonly used for these patients. In recent years, valve-sparing aortic root replacement surgery is preferred in aortic root dilatation with normofunctional valves. Performing AAR+AVR in patients with bicuspid aortic valve (BAV) do not completely remove the underlying pathology, since the defect remains in the tunica media of the aorta. The aim of this study is to investigate the change in the dimension of sinus of Valsalva in patients who underwent supracoronary AAR together with AVR.

## METHODS

### Study Design and Patient's Population

This is a retrospective study of 81 patients who underwent AAR+AVR for AAA and aortic valve diseases. After receiving approval of the Institutional Ethics Committee of our hospital, we retrospectively reviewed the medical records of patients who underwent AAR+AVR at Kartal Koşuyolu Yuksek Ihtisas Education and Research Hospital. Patients who underwent additional procedures were excluded. All preoperative, intraoperative and postoperative data were collected. The status of the patients was determined through telephone interviews and the examination of patients' cards. During follow-up, 10 (12.4%) of 81 patients died. The causes of late death in these patients were myocardial infarction, cerebrovascular accident and colorectal cancer.

The patients were divided into three groups according to the aortic valve diseases. Group I (n=17) included patients with bicuspid valves, group II (n=30) was made up of patients with stenotic degenerative valves, and patients with aortic valve regurgitation constituted group III (n=24). The mean follow-up periods were 46.59±12.64 months, 69.20±38.42 months and 58.50±23.29 months for groups I, II and III, respectively. Preoperative findings of the patients are summarized in Table 1. Of the 81 patients, 53 (65.4 %) were male and the mean age was 52.84±15.53 years. Twelve (14.8%) patients were in New York Heart Association (NYHA) functional class III and there were two patients with poor left ventricular function.

**Table 1:** Demographic characteristics.

Preoperative variables	(n=81)
Age (years/mean±SD)	54.1±15.1
Sex (female), n (%)	28 (34.6%)
Height (cm/mean±SD)	165.3±9.9
Weight (kg/mean±SD)	72±13.5
NYHA III, n (%)	12 (14.8%)
Preoperative AF, n (%)	9 (11.1%)
Hypertension, n (%)	52 (64.2%)
Diabetes mellitus, n (%)	9 (11.1%)
Smoking, n (%)	35 (43.2%)
LV function (EF/%)	
Good, n (%)	55 (67.9%)
Moderate, n (%)	24 (29.6%)
Poor, n (%)	2 (2.5%)
LVESD (mm/mean±SD)	39±11
LVEDD (mm/mean±SD)	56±10
AAD (mm/mean±SD)	53±8

Data are presented as mean value±standard deviation, median value, or number of patients. AAD=ascending aorta diameter; AF=atrial fibrillation; LV=left ventricle; EF=ejection fraction; LVEDD=left ventricular end-diastolic diameter; LVESD=left ventricular end-systolic diameter; NYHA=New York Heart Association

In the follow-up period, the change in the sinus of Valsalva diameter of the patients was evaluated with echocardiographic examination. Preoperative echocardiographic data, such as ejection fraction, left ventricular end-systolic, end-diastolic diameters and diameters of ascending aorta and sinus of Valsalva were collected and evaluated (Table 1).

### Statistical Analysis

Number Cruncher Statistical System (NCSS) 2007&PASS 2008 Statistical Software (Utah, USA) was used for statistical analysis. In the evaluation of data, descriptive statistical methods (mean, standard deviation, frequency) were used. Data were analyzed by one-way ANOVA, paired samples T-test, and T-test. Chi-square and McNemar's test were used in the comparison of qualitative data. A two-tailed probability (*P*) value of <0.05 was considered to be statistically significant.

## RESULTS

Operative data are shown in Table 2. The aortic valve was replaced by a tissue valve in 4 (4.9%) patients, and a mechanical valve in 77 (95.1%) patients. The mean size of the replaced valve was 23.50±1.6 mm. All patients received a Dacron graft with a mean size of 29.3±1.6 mm.

**Table 2:** Intraoperative parameters.

Variables		(n=81)
Artery cannulation, n (%)	Distal ascending aorta	32 (39.5%)
	Femoral artery	33 (40.7%)
	Axillary artery	15 (18.5%)
	Innominate artery	1 (1.3%)
Vein cannulation, n (%)	Right atrium	79 (97.5%)
	Femoral vein	2 (2.5%)
Cardioplegia, n (%)	Antegrade	6 (7.4%)
	Retrograde	44 (54.3%)
	Antegrade and retrograde	31 (38.3%)
Prosthetic valve, n (%)	Mechanical	77 (95.1%)
	Biological	4 (4.9%)
Hypothermia, n (%)	Mild	20 (24.7%)
	Moderate	55 (67.9%)
	Deep	6 (7.4%)
TCA, n (%)	Used	16 (19.7%)
Cerebral perfusion, n (%)	Antegrade	10 (12.3%)
	Retrograde	6 (7.4%)
	Nil	65 (80.3%)
APV (mm/mean±SD)		23.5±1.6
Aortic graft size (mm/mean±SD)		29.3±1.6
TPT (min/mean±SD)		145.9±46.7
ACC (min/mean±SD)		99±36
ICU stay (days)		3.8±1.8
Hospital stay (days)		10.3±4.2

ACC=aortic cross-clamping time; APV=aortic prosthetic valve; ICU=intensive care unit; TCA=total circulatory arrest; TPT=total perfusion time

### Follow-Up

Early complications after AAR+AVR are presented in Table 3. There was no early mortality (<30 days). The mean intensive care unit and hospital length of stay were 3.8±1.8 and 10.3±4.2 days, respectively. New-onset atrial fibrillation developed in 16 (19.7%) patients and was medically resolved in all. Inotropic support over 24 hours was needed in 10 cases, and cerebrovascular accident was treated in 4 patients.

**Table 3:** Early and late morbidity and mortality.

Variables	n (%)
Early (<30 days)	
Mortality	__
New-onset atrial fibrillation	16 (19.7%)
Reoperation for bleeding	2 (2.5%)
Pleural effusion requiring drainage	8 (9.9%)
Inotropic support >24 hours	10 (12.3%)
Acute renal failure	3 (3.7%)
Cerebrovascular accident	4 (4.9%)
Permanent pacemaker implantation	1 (1.3%)
Pulmonary complications	5 (6.2%)
Superficial wound infection	2 (2.5%)
Late	
Mortality	10 (12.4%)
Cardiac	2 (2.5%)
Non-cardiac	8 (9.9%)
Reoperation	1 (2.5%)
Endocarditis	1 (2.5%)
Thromboembolism	2 (4.3%)

Data are presented as mean±SD or as number and percentage.

Late follow-up was obtained in 71 patients with a mean of 60±30.1 months postoperatively. Mortality developed in 10 (12.4%) patients, 8 (9.9%) due to non-cardiac reasons. Only one (2%) patient needed reoperation because of infective endocarditis.

Postoperative echocardiographic parameters, such as the interventricular septum, the maximum and the mean transvalvular gradient across the aortic valve, and sinotubular junction and sinus of Valsalva diameters for each group are presented in Table 4.

**Table 4:** Postoperative echocardiographic parameters.

Echocardiographic parameters	Group I (n=17)	Group II (n=30)	Group III (n=24)	*P*
IVS, (mm/mean±SD)	11.70±1.72	11.83±2.32	10.79±1.02	0.100
Max grad, (mmHg/mean±SD)	26.23±11.61	30.03±14.40	22.41±8.03	0.072
Mean grad, (mmHg/mean±SD)	14.65±8.06	16.30±8.56	12.12±5.05	0.129
Sinus of Valsalva diameter (mm/mean±SD)	37.76±6.36	36.10±5.12	37.50±4.86	0.500
Sinotubular junction diameter (mm/mean±SD)	32.53±5.58	31.03±7.17	32.87±4.32	0.491

One-way ANOVA test.

IVS=interventricular septum; Max grad=maximum gradient; Mean grad=mean gradient

There was no significant difference among the groups in the postoperative sinus of Valsalva diameter (*P*>0.05) (Table 4). The increase in postoperative sinus of Valsalva diameter compared to preoperative size in Group I was statistically significant (*P*<0.01). There was no significant change in postoperative sinus of Valsalva diameter compared to preoperative size in Groups II and III (*P*>0.05) (Table 5). The postoperative increase in the sinus of Valsalva diameter, compared to preoperative size in the Group I, was significantly higher when compared to Group II and Group III (*P*<0.01).

**Table 5:** Changes in sinus of Valsalva diameters in different groups.

Sinus of Valsalva diameter	Group I (n=17)	Group II (n=30)	Group III (n=24)	*P*^+^
Preoperative	32.76±2.13	35.80±5.26	36.96±4.93	0.018*
Postoperative	37.76±6.36	36.10±5.12	37.50±4.86	0.500
*P*^++^	0.001**	0.071	0.085	

+ One-way ANOVA test;

++ Paired sample t-test;

* *P*<0.05;

** *P*<0.01.

## DISCUSSION

There are a number of surgical strategies for patients with AAA and concomitant aortic valve diseases. When aortic root aneurysm is present, Bentall-De Bono surgery is preferred, while AVR and supracoronary AAR is used in patients with AAA and aortic valve pathologies without annular or sinus dilatation^[2,3]^. The operative outcomes of supracoronary AAR with AVR are excellent and there is no additional risk in elective and non-high-risk patients^[4]^. The aetiology of valvular disease, intraoperative shape and ascending aortic wall thickness and the patient's condition are important factors for surgical decision in BAV^[5]^.

AAA seen in aortic stenosis of calcific degeneration is usually post-stenotic dilatation. It is progressive and its rate of increase is reported to be >3 mm/year^[1]^. If aortic valve and ascending aorta were replaced in these patients, the underlying pathology would be cured. In our study, 30 patients had aortic stenosis caused by calcific degeneration. Regarding the sinus of Valsalva diameter, the postoperative diameter, compared to the preoperative size, did not change significantly (*P*>0.05). In AAA with aortic valve insufficiency, the sinus of Valsalva diameter is also enlarged. With progression of AAA, dilatation of the sinotubular junction, displacement of commissures, distortion or dilatation of one or more sinus of Valsalva, annuloaortic ectasia alone or in combination can cause aortic regurgitation. In our study, 24 patients underwent AAR+AVR due to aortic regurgitation and AAA. The postoperative sinus of Valsalva diameter, compared with preoperative size, was not significantly different (*P*>0.05). Although sinus of Valsalva was dilated preoperatively in some patients, composite valve-graft replacement or valve-sparing aortic root replacement was not performed. These operations may have been avoided because of the patients' advanced age.

BAV is not just a valvular disease, but a component of a wider pathology also including the ascending aorta^[6]^. Although tunica media and normal aortic valve in BAV is the same, the gap between elastic lamella is greater. Patients with BAV have thinner elastic lamellae of the aortic medium than patients with tricuspid aortic valve^[7]^. Fibrillin-1 is a glycoprotein needed for structural continuity of aortic wall and valves. Fibrillin-1 deficiency is more common in BAV compared with that seen in tricuspid aortic valves^[8]^. Higher activity of proteolytic enzymes known as matrix metalloproteins was seen in aortic aneurysms associated with BAV when compared to aneurysms of patients with tricuspid valves^[9]^. High tension and shear stress play an important role in the pathogenesis of ascending aortic aneurysm with BAV. Tensile stress is the force perpendicular to the aortic wall, and increases with aortic diameter according to the law of Laplace. On the other hand, shear stress is a product of blood viscosity and velocity and is a force parallel to the aortic wall causing friction to the endothelial surface^[6]^. Current guidelines suggest the replacement of the ascending aorta in the presence of a diameter of 50 mm or more, if associated with BAV disease with additional risk factors or coarctation^[5]^.

In our study, AAR+AVR was performed in 17 patients with BAV and AAA. Postoperative sinus of Valsalva diameter, compared with the preoperative size, was significantly increased in these patients (P<0.01). The follow-up period of these patients was 46.59±12.64 months.

The relationship between follow-up duration and sinus of Valsalva diameter was not statistically significant. It could be due to a small numbers of patients and shorter follow-up in some patients. An increase of 5±4.63 mm in sinus of Valsalva diameter was noted in this short follow-up. Longer follow-up duration in a larger group of patients could have revealed a significant increase in sinus of Valsalva diameter because the disease is progressive. Although the evolution of the ascending aorta with BAV is well documented in many studies^[10,11]^, the risk of progressive sinus of Valsalva dilatation is less clear. Vendramin et al.^[12]^ showed that no progressive sinus Valsalva dilatation is recorded in the long-term follow-up. Conversely, a significant reduction of the mean aortic root diameter was observed in some patients.

According to Yasuda et al.^[13]^, BAV replacement, either by stenosis or failure, did not prevent the progressive dilation of the proximal aorta, which differs from that observed in patients with tricuspid aortic valve.

Russo et al.^[14]^, when following-up more than 100 patients undergoing AVR, also reported a higher incidence of sudden death and aortic dissection in a group of patients with BAV, a significantly larger increase in aortic diameter in the same group, suggesting that prophylactic surgery for replacement of the ascending aorta concomitant with valve replacement should be performed, even in the presence of mild dilation of the ascending aorta.

Borger et al.^[15]^, in a clinical retrospective study assessing the aortic complications in patients with BAV, concluded that patients with aortic diameter exceeding 45 mm should undergo combined surgery, or that is, AVR and replacement of the ascending aorta to avoid reinterventions due to vascular complications, either aneurysms and dissections of the ascending aorta. In another study, there were no late reoperations for aortic root dissection or rupture in 124 BAV patients who underwent AAR+AVR during a follow-up of 75.2 months^[16]^.

Study published by Etz et al.^[17]^ reported that in cases of surgery due to aortic valve disease, associated with a diameter of the root or ascending aorta exceeding 4.0 cm and life expectancy greater than 10 years, the option was to replace both the valve and the aorta.

Some studies have shown that sinus of Valsalva aneurysm developed in long-term follow-up of patients who underwent AAR+AVR because of type A dissection^[18,19]^. In our hospital, valve-sparing aortic root replacement or composite valve-graft replacement was performed in patients with type A dissection. That is the reason why we did not include these patients to our study.

In a study by Yun et al.^[20]^, 49 of the 255 patients who underwent AAR+AVR were reoperated for aortic root pathologies. In a study by Houël et al.^[21]^, the rate of freedom from second operation for aortic root pathologies was 97.3±1.9% in composite valve-graft replacement and 68.3±9% in AAR+AVR. It was stated that AAR+AVR was a risk factor for complications related to the aortic root. When these two studies were compared to our study, the rate of complications related to the aortic root was lower in our study. We included 71 patients and no patient was reoperated for aortic root pathologies in the medium-term follow-up. We assumed that this success was due to the choice of proper surgical technique and exclusion of dissection.

### Limitations

The major limitation of this study is its retrospective nature, spanning 10 years and involving a limited number of patients. A potential limitation of our study is the short duration of follow-up in some patients.

## CONCLUSION

In patients with ascending aortic aneurysm and concomitant aortic valve diseases, the surgical technique should be selected according to the underlying disease. To avoid the risks associated with sinus of Valsalva dilatation, it is reasonable to replace the sinus of Valsalva in the setting of AVR and AAR for BAV with a dilated ascending aorta and relatively normal sinuses of Valsalva in young patients.

**Table:** 

**Authors’ roles & responsibilities**	
SS	Substantial contributions to the conception or design of the work; or the acquisition, analysis, or interpretation of data for the work; drafting the work or revising it critically for important intellectual content; agreement to be accountable for all aspects of the work in ensuring that questions related to the accuracy or integrity of any part of the work are appropriately investigated and resolved; final approval of the version to be published
EC	Drafting the work or revising it critically for important intellectual content; final approval of the version to be published
CK	Substantial contributions to the conception or design of the work; or the acquisition, analysis, or interpretation of data for the work; agreement to be accountable for all aspects of the work in ensuring that questions related to the accuracy or integrity of any part of the work are appropriately investigated and resolved; final approval of the version to be published
HMA	Agreement to be accountable for all aspects of the work in ensuring that questions related to the accuracy or integrity of any part of the work are appropriately investigated and resolved; final approval of the version to be published
